# How to get started in quality improvement

**DOI:** 10.1136/bmj.k5437

**Published:** 2019-01-17

**Authors:** Bryan Jones, Emma Vaux, Anna Olsson-Brown

**Affiliations:** 1 The Health Foundation, London, UK; 2 Royal Berkshire NHS Foundation Trust. Reading, UK; 3 Department of Molecular and Clinical Pharmacology, The Institute of Translational Medicine, University of Liverpool, Liverpool, UK

**What you need to know**

- Participation in quality improvement can help clinicians and trainees improve care together and develop important professional skills
- Effective quality improvement relies on collaborative working with colleagues and patients and the use of a structured method
- Enthusiasm, perseverance, good project management skills, and a willingness to explain your project to others and seek their support are key skills

Quality improvement (box 1) is a core component of many undergraduate and postgraduate curriculums.^1^ ^2^ ^3^ ^4^ ^5^ Numerous healthcare organisations,^6^ professional regulators,^7^ and policy makers^8^ recognise the benefits of training clinicians in quality improvement.

Box 1 **Defining quality improvement^1^**

Quality improvement aims to make a difference to patients by improving safety, effectiveness, and experience of care by:
Using understanding of our complex healthcare environment
Applying a systematic approach
Designing, testing, and implementing changes using real time measurement for improvement

Engaging in quality improvement enables clinicians to acquire, assimilate, and apply important professional capabilities^7^ such as managing complexity and training in human factors.^1^ For clinical trainees, it is a chance to improve care^9^; develop leadership, presentation, and time management skills to help their career development^10^; and build relationships with colleagues in organisations that they have recently joined.^11^ For more experienced clinicians, it is an opportunity to address longstanding concerns about the way in which care processes and systems are delivered, and to strengthen their leadership for improvement skills.^12^

The benefits to patients, clinicians, and healthcare providers of engaging in quality improvement are considerable, but there are many challenges involved in designing, delivering, and sustaining an improvement intervention. These range from persuading colleagues that there is a problem that needs to be tackled, through to keeping them engaged once the intervention is up and running as other clinical priorities compete for their attention.^13^ You are also likely to have competing priorities and will need support to make time for quality improvement. The organisational culture, such as the extent to which clinicians are able to question existing practice and try new ideas,^14^ ^15^ ^16^ also has an important bearing on the success of the intervention.

This article describes the skills, knowledge, and support needed to get started in quality improvement and deliver effective interventions.

## What skills do you need?

Enthusiasm, optimism, curiosity, and perseverance are critical in getting started and then in helping you to deal with the challenges you will inevitably face on your improvement journey.

Relational skills are also vital. At its best quality improvement is a team activity. The ability to collaborate with different people, including patients, is vital for a project to be successful.^17^ ^18^ You need to be willing to reach out to groups of people that you may not have worked with before, and to value their ideas.^19^ No one person has the skills or knowledge to come up with the solution to a problem on their own.

Learning how systems work and how to manage complexity is another core skill.^20^ An ability to translate quality improvement approaches and methods into practice (box 2), coupled with good project and time management skills, will help you design and implement a robust project plan.^27^

Box 2 **Quality improvement approaches**

Healthcare organisations use a range of improvement methods,^21^ ^22^ such as the Model for Improvement, where changes are tested in small cycles that involve planning, doing, studying, and acting (PDSA),^23^ and Lean, which focuses on continually improving processes by removing waste, duplication, and non-value adding steps.^24^ To be effective, such methods need to be applied consistently and rigorously, with due regard to the context.^25^ In using PDSA cycles, for example, it is vital that teams build in sufficient time for planning and reflection, and do not focus primarily on the “doing.”^26^

Equally important is an understanding of the measurement for improvement model, which involves the gradual refinement of your intervention based on repeated tests of change. The aim is to discover how to make your intervention work in your setting, rather than to *prove* it works, so useful data, not perfect data, are needed.^28^ ^29^ Some experience of data collection and analysis methods (including statistical analysis tools such as run charts and statistical process control) is useful, but these will develop with increasing experience.^30^ ^31^

Most importantly, you need to enjoy the experience. It is rare that a clinician can institute real, tangible change, but with quality improvement this is a real possibility, which is both empowering and satisfying. Finally, don’t worry about what you don’t know. You will learn by doing. Many skills needed to implement successful quality improvement will be developed as you go; this is a fundamental feature of quality improvement.

## How do you get started?

The first step is to recruit your improvement team. Start with colleagues and patients,^32^ but also try to bring in people from other professions, including non-clinical staff. You need a blend of skills and perspectives in your team. Find a colleague experienced in quality improvement who is willing to mentor or supervise you.

Next, identify a problem collaboratively with your team. Use data to help with this (eg, clinical audits, registries of data on patients’ experiences and outcomes, and learning from incidents and complaints) (box 3). Take time to understand what might be causing the problem. There are different techniques to help you (process mapping, five whys, appreciative inquiry).^35^ ^36^ ^37^ Think about the contextual factors that are contributing to the problem (eg, the structure, culture, politics, capabilities and resources of your organisation).

Box 3 **Clinical audit and quality improvement**

Quality improvement is an umbrella term under which many approaches sit, clinical audit being one.^33^ Clinical audit is commonly used by trainees to assess clinical effectiveness. Confusion of audit as both a term for assurance and improvement has perhaps limited its potential, with many audits ending at the data collection stage and failing to lead to improvement interventions. Learning from big datasets such as the National Clinical Audits in the UK is beginning to shift the focus to a quality improvement approach that focuses on identifying and understanding unwanted variation in the local context; developing and testing possible solutions, and moving from one-off change to multiple cycles of change.^34^

Next, develop your aim using the SMART framework: Specific (S), Measurable (M), Achievable (A), Realistic (R), and Timely (T).^38^ This allows you to assess the scale of the intervention and to pare it down if your original idea is too ambitious. Aligning your improvement aim with the priorities of the organisation where you work will help you to get management and executive support.^39^

Having done this, map those stakeholders who might be affected by your intervention and work out which ones you need to approach, and how to sell it to them.^40^ Take the time to talk to them. It will be appreciated and increases the likelihood of buy in, without which your quality improvement project is likely to fail irrespective of how good your idea is. You need to be clear in your own mind about the reasons you think it is important. Developing an “elevator pitch” based on your aims is a useful technique to persuade others,^38^ remembering different people are hooked in for different reasons.

The intervention will not be perfect first time. Expect a series of iterative changes in response to false starts and obstacles. Measuring the impact of your intervention will enable you to refine it.^28^ Time invested in all these aspects will improve your chances of success.

Right from the start, think about how improvement will be embedded. Attention to sustainability will mean that when you move to your next job your improvement efforts, and those of others, and the impact you have collectively achieved will not be lost.^41^ ^42^

## What support is needed?

You need support from both your organisation and experienced colleagues to translate your skills into practice. Here are some steps you can take to help you make the most of your skills:

- Find the mentor or supervisor who will help identify and support opportunities for you. Signposting and introduction to those in an organisation who will help influence (and may hinder) your quality improvement project is invaluable
- Use planning and reporting tools to help manage your project, such as those in NHS Improvement’s project management framework^27^
- Identify if your local quality improvement or clinical audit team may be a source of support and useful development resource for you rather than just a place to register a project. Most want to support you.
- Determine how you might access (or develop your own) local peer to peer support networks, coaching, and wider improvement networks (eg, NHS networks; Q network^43^ ^44^)
- Use quality improvement e-learning platforms such as those provided by Health Education England or NHS Education for Scotland to build your knowledge^45^ ^46^
- Learn through feedback and assessment of your project (eg, via the QIPAT tool^47^ or a multi-source feedback tool.^48^ ^49^

Quality improvement approaches are still relatively new in the education of healthcare professionals. Quality improvement can give clinicians a more productive, empowering, and educational experience. Quality improvement projects allow clinicians, working within a team, to identify an issue and implement interventions that can result in true improvements in quality. Projects can be undertaken in fields that interest clinicians and give them transferable skills in communication, leadership, project management, team working, and clinical governance. Done well, quality improvement is a highly beneficial, positive process which enables clinicians to deliver true change for the benefit of themselves, their organisations, and their patients.

**Quality improvement in action: three doctors and a medical student talk about the challenges and practicalities of quality improvement**

This box contains four interviews by Laura Nunez-Mulder with people who have experience in quality improvement.

**Alex Thompson, medical student at the University of Cambridge, is in the early stages of his first quality improvement project**

We are aiming to improve identification and early diagnosis of aortic dissections in our hospital. Our supervising consultant suspects that the threshold for organising computed tomography angiography for a suspected aortic dissection is too high, so to start with, my student colleague and I are finding out what proportion of CT angiograms result in a diagnosis of aortic dissection.

I fit the project around my studies by working on it in small chunks here and there. You have to be very self motivated to see a project through to the end.

**Anna Olsson-Brown, research fellow at the University of Liverpool, engaged in quality improvement in her F1 year, and has since supported junior doctors to do the same. This extract is adapted from her *BMJ Opinion* piece (https://blogs.bmj.com/bmj/)**

Working in the emergency department after my F1 job in oncology, I noticed that the guidelines on neutropenic sepsis antibiotics were relatively unknown and even less frequently implemented. A colleague and I devised a neutropenic sepsis pathway for oncology patients in the emergency department including an alert label for blood tests. The pathway ran for six months and there was some initial improvement, but the benefit was not sustained after we left the department.

As an ST3, I mentored a junior doctor whose quality improvement project led to the introduction of a syringe driver prescription sticker that continues to be used to this day.

My top tips for those supporting trainees in quality improvement:

- Make sure the project is sufficiently narrow to enable timely delivery
- Ensure regular evaluation to assess impact
- Support trainees to implement sustainable pathways that do not require their ongoing input.

**Amar Puttanna, consultant in diabetes and endocrinology at Good Hope Hospital, describes a project he carried out as a chief registrar of the Royal College of Physicians**

The project of which I am proudest is a referral service we launched to review medication for patients with diabetes and dementia. We worked with practitioners on the older adult care ward, the acute medical unit, the frailty service, and the IT teams, and we promoted the project in newsletters at the trust and the Royal College of Physicians.

The success of the project depended on continuous promotion to raise awareness of the service because junior doctors move on frequently. Activity in our project reduced after I left the trust, though it is still ongoing and won a Quality in Care Award in November 2018.

Though this project was a success, not everything works. But even the projects that fail contain valuable lessons.

**Mark Taubert, consultant in palliative medicine and honorary senior lecturer for Cardiff University School of Medicine, launched the TalkCPR project**

Speaking to people with expertise in quality improvement helped me to narrow my focus to one question: “Can videos be used to inform both staff and patients/carers about cardiopulmonary resuscitation and its risks in palliative illness?” With my team I created and evaluated TalkCPR, an online resource that has gone on to win awards (talkcpr.wales).

The most challenging aspect was figuring out which tools might get the right information from any data I collected. I enrolled on a Silver Improving Quality Together course and joined the Welsh Bevan Commission, where I learned useful techniques such as multiple PDSA (plan, do, study, act) cycles, driver diagrams, and fishbone diagrams.

**Education into practice**

In designing your next quality improvement project:

- What will you do to ensure that you understand the problem you are trying to solve?
- How will you involve your colleagues and patients in your project and gain the support of managers and senior staff?
- What steps will you take right from the start to ensure that any improvements made are sustained?

**How patients were involved in the creation of this article**

The authors have drawn on their experience both in partnering with patients in the design and delivery of multiple quality improvement activities and in participating in the Academy of Medical Royal Colleges Training for Better Outcomes Task and Finish Group^1^ in which patients were involved at every step. Patients were not directly involved in writing this article.

**Sources and selection material**

Evidence for this article was based on references drawn from authors’ academic experience in this area, guidance from organisations involved in supporting quality improvement work in practice such as NHS Improvement, The Health Foundation, and the Institute for Healthcare Improvement, and authors’ experience of working to support clinical trainees to undertake quality improvement.

## Notes

Provenance and peer review: This article is part of a series commissioned by The BMJ based on ideas generated by a joint editorial group with members from the Health Foundation and The BMJ, including a patient/carer. The BMJ retained full editorial control over external peer review, editing, and publication. Open access fees and The BMJ’s quality improvement editor post are funded by the Health Foundation.
